# Application of a geospatial query tool to characterise the community food environment and examine associations with dietary quality: evidence from three Chilean cities from the SALURBAL project

**DOI:** 10.1186/s12889-025-23392-x

**Published:** 2025-07-03

**Authors:** Laís Vargas Botelho, Dayan Carvalho Ramos Salles de Oliveira, Amy Auchincloss, Irene Carolina Sousa Justiniano, Maria de Fátima de Pina, Vanderlei Pascoal de Matos, Daniel Albert Skaba, Lorena Saavedra-Garcia, Tamara Doberti Herrera, Letícia de Oliveira Cardoso, Mariana Carvalho de Menezes

**Affiliations:** 1 National School of Public Health, Oswaldo Cruz Foundation, RJ 21041-210 Rio de Janeiro, Brazil; 2 Department of Epidemiology and Biostatistics and Urban Health Collaborative, Dornsife School of Public Health, Drexel University, Philadelphia, PA USA; 3 School of Nutrition, Federal University of Minas Gerais, Belo Horizonte, 35.400-000 MG Brazil; 4 CRONICAS Center of Excelence in Chronic Diseases, Cayetano Heredia University, Lima, 15074 Peru; 5 University of Chile, Santiago, 939 Chile

**Keywords:** Community food environment, Food retail, Urban health, Chile, Latin America, Dietary quality

## Abstract

**Background:**

Few studies have characterised community food environments and their influence on dietary behaviours in Latin America. In particular, community food environment data do not exist for Chilean contexts. This study aims to characterise the community food environment across neighbourhoods in three major Chilean cities and explore associations between these food environments and fruit and vegetable consumption among adult residents.

**Methods:**

We used a geospatial query tool to identify, georeference, and classify food establishments (FE, *n* = 46,950) across 3 large Chilean cities (Santiago, Valparaíso, Concepción). Neighbourhood characteristics were derived from the 2017 Census (*n* = 2,442 neighbourhoods) while individual-level data came from the 2017 National Health Survey (Encuesta Nacional de Salud, *n* = 1,275 adults). Lower dietary quality was proxied by non-daily consumption of fruits or vegetables (ND-FV, prevalence 17.3%). We used random intercept logistic regression models adjusting for survey participants age, gender, educational attainment, neighbourhood population density and social environment.

**Results:**

The median density of FE was 41.36 per km^2^, increasing with neighbourhood population density and more favourable social environments. Ready-for-consumption FE and small food retail were the most prevalent types (68% and 11%, respectively). The adjusted odds of ND-FV consumption was approximately 20% higher with increases in the z-score of those FE types. No associations were found between other types of FE and ND-FV consumption.

**Conclusion:**

Food establishments were unequally distributed across urban Chilean neighbourhoods. Overall, unhealthy outlets are predominant. Higher densities of small and ready-to-consume FE were associated with lower fruit and vegetable consumption. These findings underscore the need for targeted public policies aimed at promoting healthier food environments, reducing social inequalities in food access within Chilean cities, and improving dietary quality.

**Supplementary Information:**

The online version contains supplementary material available at 10.1186/s12889-025-23392-x.

## Background

A growing body of evidence suggests that physical, social, economic, and political factors significantly influence how people obtain and consume food, a concept encapsulated in the term “food environment” [1]. There is fairly strong evidence demonstrating that food availability influences dietary behaviours in high-income countries [2–7]. However, much less research has been done in Latin American contexts, leaving knowledge gaps on food environments—particularly the community food environment, which encompasses the accessibility of food retailers (e.g., grocery stores and markets) [1]. Additionally, associations between the food environment and dietary practices remain understudied in this region [4

Findings from high-income contexts may not be transportable to Latin America due to the differences in food retail types and its spatial distribution, target populations reached by each establishment, cultural relevance of diverse food retail formats, and criteria adopted in studies to define food healthiness [8–13]. In the present study, we define a healthy diet as one primarily based on fresh or minimally processed foods and a low intake of ultra-processed foods [9]. Since food environment studies often classify establishments based on product healthiness, such definitions are pivotal for cross-context comparisons.

In high-income countries, supermarkets, grocery stores and convenience stores are common. While supermarkets offer both healthy and unhealthy foods, they also serve as the primary source of fresh and minimally processed foods and are often considered a proxy for healthy food availability [8]. In contrast, in Latin American countries, supermarkets are primarily characterised by their offering of affordable ultra-processed products [9, 10], and household food purchase is split among retail outlets such as supermarkets, traditional markets, specialised stores (i.e. fruit shops), and small local shops (i.e. “tiendas de abarrotes” or “bodegas”) [11]. In addition, traditional markets, small local shops, and street vendors—individuals or small businesses selling food in public spaces using simple structures such as pushcarts, bicycles, wheelbarrows, or simple stalls—are culturally relevant, as they are part of the gastronomic culture of Latin American countries [12, 13].

However, in recent decades, significant changes have occurred in the community food environment globally [14, 15] and in Latin American contexts specifically [9, 14–16]. Smaller food establishments are losing ground to larger international food companies [9, 14, 15] leading to more ultra-processed foods (UPF), such as chips and sugar-sweetened beverages, being commercialised [9]. This has made these unhealthy foods more available in local communities, characterizing a transition to less healthy diets with wide variations across regions and countries [9]. This shift has health implications, as higher consumption of UPF is associated with a higher risk of overweight and obesity, all-cause mortality, metabolic syndrome, and depression in adults [17].

A major barrier to studying the community food environment is the difficulty of gathering relevant data. Collecting direct field data over large areas is costly, and in most Latin American contexts, a comprehensive data source of food establishments does not exist [4]. Research on this topic in the region has mostly been small-scale, focused on limited sections of cities [3, 18] and typically requires observation data, such as those obtained from in-store audit tools [18].

To address this limitation, we developed an innovative tool designed to automatically capture food retail data from Google Earth (GE). The tool demonstrated moderate to excellent validity [19]. The aims of this current study are: [1] to apply the tool to extract information about the community food environment in three large Chilean cities; [2] characterise the community food environment across neighbourhoods; [3] link the food environment data to a population-based surveillance survey that collected data on fruit and vegetable (FV) consumption; and [4] explore possible associations between the community food environment and FV consumption among adults.

## Methods

### Study spatial units

The present study used data from three Chilean cities: Santiago, Valparaíso, and Concepción. The three cities were chosen based on their large population, high population density, and high tourism and/or retail activity– characteristics under which the web data mining tool we used to extract retail food information performed well in prior studies [19]. Within the three cities, there were a total of 2,442 *zona censales*. *Zonas censales* are analogous to US census tracts, each of which includes, on average, approximately 700 households, and were used as proxies for ‘neighbourhood’ units.

This cross-sectional study used secondary data and was conducted as part of the SALURBAL project (SALud URBana en América Latina—Urban Health in Latin America) [20]. The SALURBAL study protocol was approved by the Drexel University Institutional Review Board (IRB) with ID #1,612,005,035, and by appropriate site-specific IRBs.

### Community food environment data

We obtained information on the community food environment using data from Google’s map between August and November 2021. Using a query tool based on Google Cloud services, we automatically collected information on registered food retail outlets located within specific points of interest (POIs) across designated cities. The algorithm searches for points of interest (POIs) based on predefined terms (such as ‘supermarket,’ ‘ice cream shop,’ and ‘bakery’), applying this search across a grid that covers the entire study area. If a given term yields no results at a specific location, the algorithm automatically moves to the next point, repeating the process until the entire area has been covered. This process returns a list of food retailers located within the selected areas, including their names, addresses, geographic coordinates, and retail types—as classified by Google—with each establishment assigned one primary and up to seven secondary categories [19].

The tool then applies a programmatic procedure to clean and de-duplicate the data. Then, it classifies the establishments into seven food outlet categories: fruit and vegetable stores, fresh food retail, small food retail, supermarkets, ready-to-eat food outlets, convenience stores, fast food chains, and candy/ice cream shops. The seven food outlet categories are also grouped into three health-related categories: most healthy, mixed, and less healthy. Additional File 1 provides detailed definitions and classification criteria for each group.

Stores in the final dataset are assigned to one of the seven categories primarily based on search terms found in their names, which are the preferred source of classification. When the name alone does not provide sufficient information, the classification defaults to the food retailer type registered in Google’s database. Establishments that could not be assigned to a category through either method were manually reviewed; those whose names and Google classifications did not match any predefined search terms or synonyms remained unclassified.

Because the accuracy of the classification depends on the outlets’ names and the terms used in data acquisition, we selected search terms from a previously developed qualitative inventory of food outlet terminology commonly used in Latin America [21]. Our tool cannot detect unregistered or no longer operational outlets; however, the algorithm’s validity was confirmed in two of Brazil’s largest cities through ground-truth observations, with sensitivity, specificity, positive predictive value, negative predictive value, and accuracy ranging from 66.3 to 100%, with no differences across neighbourhood socioeconomic strata [19].

Our query tool returned a list of 48,400 food retailers. Less than 3% of the food establishments that we compiled could not be successfully classified (*n* = 1,450). These establishments were retained in the total but are not represented when data are stratified by food establishment type (see Additional file 2). Since street markets and street food vendors are not considered permanent outlets and are not listed on Google, they were excluded from the analysis.

Finally, food establishments densities were calculated at the neighbourhood level by dividing the number of food establishments per square kilometre area within each neighbourhood.

### Neighbourhood census characteristics: population and social environment data

We used data from the 2017 Chilean census to establish neighbourhood population density and evaluate the social environment. These variables are essential when studying the connections between food environment characteristics and eating patterns. Previous research has noted that neighbourhoods with higher population density tend to have a denser availability of food stores in Latin American countries [4, 22, 23]. Similarly, the availability of total food stores, along with the distribution of specific types, varies based on the neighbourhood’s socioeconomic status [3, 24, 25].

Population density was calculated by dividing the population by the neighbourhood area (km²). To characterise the neighbourhood’s social environment, we used a summary index developed for the SALURBAL study, as detailed elsewhere [26]. This index integrated variables related to sanitation, housing, employment, and education dimensions available for the neighbourhoods in our study. Variables were converted to z-scores and subsequently averaged. Higher values indicate a more favourable social environment within a neighbourhood.

### Individual data

Individual level survey data came from year 2017 Encuesta Nacional de Salud (ENS, National Health Survey), a cross-sectional survey that is nationally representative of households in Chile. The survey is administered primarily in person and collects information on a wide range of health behaviours and outcomes for surveillance and planning purposes. There were 1,292 adults (aged ≥ 20) who completed the survey in the three cities. We used survey variables for age, gender, educational level (incomplete high school, completed high school or above), and FV consumption. We excluded 17 individuals missing one or more of these variables, leaving 1,275 in the individual-level sample (see Additional file 2).

### Dietary measures

FV consumption information was collected through the following ENS 2017 questions: “In a typical week, how many days do you eat fruits (response options 0–7)?” and “In a typical week, how many days do you eat vegetables or vegetable salads (response options 0–7)? Don’t include potatoes or legumes.”. These variables were summed and capped at 7 days to create a new variable corresponding to the number of days per week that FV were consumed. Additionally, we created a binary variable that reflected non-daily consumption of FV vs. daily, serving as a proxy for lower dietary quality. Participants were assigned to FV ‘non-daily’ if they reported consuming FV fewer than 7 days a week (vs ‘daily,’ which was 7 days a week).

### Statistical analyses

Descriptive statistics are presented for neighbourhood census characteristics and food establishments densities. We present the distribution of the neighbourhood data within each city, mean/median food establishment densities by census characteristics and the proportion of the different types of establishments investigated. Survey data on non-daily vs. daily consumption of FV was presented by demographic characteristics and food establishment’s densities and census characteristics.

To estimate the odds of non-daily FV consumption (outcome) according to food establishment’s densities (exposures), we used two-level mixed effect logistic regression models of individuals nested within neighbourhoods (random intercept for neighbourhoods). Model Akaike Information Criterion (AIC) guided our decisions regarding the best structure for modelling the nested structure of the data. We used a progressive modelling strategy to better understand how adjustment affects the association between food retail types and daily FV consumption (Table 5). We fitted three models for each food establishment exposure: [1] unadjusted single exposure model; [2] adjusted by person-level confounders (age, gender, and individual educational level); and [3] model 2 variables plus census characteristics (population density and intra urban social environment index). To aid interpretation, all independent variables were standardised to a mean of zero and a standard deviation of 1 (z-score).

All analyses were conducted in R programming language, version 4.2.2.

## Results

### Neighbourhood characteristics

Among the three cities, Santiago had the highest population density (12,494 people/km^2^), in contrast to Valparaíso and Concepción (6,802 and 5,079 people/km^2^, respectively, Table 1). Santiago also had a more favourable neighbourhood social environment score (0.46, compared to vs. 0.13 and − 0.23 for Valparaíso and Concepción, respectively).

**Table 1:** Neighbourhood census characteristics, total and by city, Chile, 2017 (*n* = 2,442 neighbourhoods)

		Overall	Santiago	Valparaíso	Concepción
**Number of neighbourhood s** ^**a**^		2,442	1,700	386	356
**Population density (number of people / km** ^**2**^ **)**					
Median		10,365	12,494	6,802	5,079
25th-75th percentile		5,791–15,252	8,100–17,535	3,814–9,759	3,814–9,759
**Social environment index (z-score)**					
Median		0.31	0.46	0.13	-0.23
25th-75th percentile		-0.17–0.85	0.01–0.96	-0.47–0.62	-0.81–0.53

^a^ Neighbourhoods refer to Chilean ‘zona censales’. The social environment index is a summary measure based on variables related to four dimensions (sanitation, housing, employment, education) assessed at the ‘zona censal’ level. Higher values indicate a better social environment within neighbourhoods

### Food establishments densities

We identified and georeferenced 48,400 establishments and were able to classify 46,950 of them (97% of all establishments). The majority (66.01%) of the classified establishments were ready-for-consumption food establishments, followed by small food stores (10.76%), convenience establishments (7.47%), supermarkets (6.97%), fresh food stores (2.13%), candy and ice cream shops (1.68%), FV stores (1.01%), and fast food chains (0.96%). Most of the establishments were in the health-related category ‘mixed’ (83.74%) with only 3.14% in the category ‘most healthy’ (a category that included FV establishments and fresh food retail) (see Additional file 3).

Patterns in neighbourhood food establishment density aligned with population density, with Santiago being the highest (56 establishments/km^2^) compared to less than 15 establishments/km^2^ in the other two cities (Tables 2 and 3). Density of the ‘most healthy’ establishments was very low, with most neighbourhoods lacking a FV or fresh food establishment. Compared to Santiago, Valparaíso and Concepción had notably lower densities of “less healthy” food establishments—a category that included chain convenience establishments, which was the third most common type of establishment in Santiago (Table 2).

**Table 2:** Retail food environment density at the neighbourhood-level (*n* = 2,442) by city, Chile, 2017

			Total	Santiago	Valparaíso	Concepción
**Number of food establishments**			48,400	38,255	4,962	5,193
**Food establishments density (count / km** ^**2**^ **)**						
	**Total** ^**a**^					
		Median	41.36	56.00	11.50	14.80
		25th-75th percentile	15.30–73.55	32.20–87.20	0–30.40	4.02–34.00
	**Most Healthy**					
	Fruit and vegetables stores					
		median	0.00	0.00	0.00	0.00
		25th-75th percentile	0–0	0–0	0–0	0–0
	Fresh food retail					
		median	0.00	0.00	0.00	0.00
		25th-75th percentile	0–1.36	0–2.17	0–0	0–0
	**Mixed**					
	Small food retail					
		median	4.10	5.08	0.49	2.23
		25th-75th percentile	0–8.91	0.89–9.86	0–6.05	0–5.91
	Supermarkets					
		median	2.41	3.48	0.00	0.61
		25th-75th percentile	0–6.11	0–7.28	0–2.39	0–3.67
	Ready-for-consumption food retail					
		median	23.19	32.30	5.60	7.85
		25th-75th percentile	7.92–47.61	16.40–56.00	0–17.30	1.84–17.50
	**Less Healthy**					
	Candy and ice cream shops					
		median	0.00	0.00	0.00	0.00
		25th-75th percentile	0–0	0–0.74	0–0	0–0
	Fast-food chains					
		median	0.00	0.00	0.00	0.00
		25th-75th percentile	0–0	0–0	0–0	0–0
	Chain convenience stores					
		median	2.37	4.51	0.00	0.00
		25th-75th percentile	0–8.04	0–10.30	0–1.23	0–1.28

^a^ The row for ‘total’ includes all classifiable establishments *n* = 46,950 + unclassifiable establishments *n* = 1,450

**Table 3:** Food establishments density at the neighboorhood-level (*n* = 2,442) by population census characteristics, Chile, 2017

		Population density/urbanicity density (per area km^2^)				Intraurban social environment index (z-score)			
		Q 1 (low)	Q 2	Q 3	Q 4 (high)	Q 1 (low)	Q 2	Q 3	Q 4 (high)
**Food establishments density (establishments/km** ^**2**^ **)**									
**Total** ^a^									
	mean	8.64	11.10	13.20	15.20	26.7	52.2	64.9	134.00
	median	4.32	15.80	15.80	15.80	16.2	45.3	53.7	61.6
**Most Healthy**									
Fruit and vegetable stores									
	mean	0.27	0.50	0.77	0.85	0.32	0.64	0.74	0.69
	median	0.00	0.00	0.00	0.00	0.00	0.00	0.00	0.00
Fresh food retail									
	mean	0.59	1.10	1.57	1.90	0.72	1.25	1.61	1.58
	median	0.00	0.00	0.00	0.00	0.00	0.00	0.00	0.00
**Mixed**									
Small food retail									
	mean	2.77	6.30	6.90	10.00	3.83	6.28	7.10	8.74
	median	0.98	4.60	5.39	7.20	1.89	4.76	4.89	4.41
Supermarkets chains									
	mean	1.53	3.49	4.97	8.66	2.59	4.32	4.94	6.80
	median	0.30	2.51	3.52	4.98	0.00	2.58	3.36	2.85
Ready-for-consumption food retail									
	mean	19.50	37.10	52.00	81.00	15.10	30.70	40.90	103.00
	median	4.94	23.20	30.40	39.00	8.59	23.00	30.20	42.30
**Less Healthy**									
Candy and ice cream shops									
	mean	0.49	0.83	1.22	1.99	0.45	0.68	1.02	2.37
	median	0.00	0.00	0.00	0.00	0.00	0.00	0.00	0.00
Fast-food chains									
	mean	0.41	0.46	0.59	1.22	0.04	0.20	0.46	1.97
	median	0.00	0.00	0.00	0.00	0.00	0.00	0.00	0.00
Chain convenience stores									
	mean	1.24	3.73	6.14	11.70	2.73	6.29	6.23	7.56
	median	0.00	2.31	4.40	8.49	0.00	3.95	4.19	2.30

^a^ The row for ‘total’ includes all classifiable establishments *n* = 46,950 + unclassifiable establishments *n* = 1,450

Q = Quartile

Food establishment densities also tended to be higher in areas with more favourable social environment index. For example, the median density per km^2^ of all food establishments was 61.6 and 16.2 in highest and lowest quartiles of social environment, respectively (Table 3). Within the food establishment categories, the pattern was most evident for ready-for-consumption establishments: median density per km^2^ was 42.3 and 8.59 for highest and lowest quartiles of social environment.

### Survey participant residential environment and FV consumption

Table 4 shows results for individual-level analyses using the survey data (*n* = 1,275). On average, the sample was 61.6% female and median age 50 years. The majority (56.2%) had less than secondary school education. Respondents lived in cities with a median population density of approximately 9,810 persons per square kilometre and a median social environment z-score 0.2.

**Table 4:** Individual (*n* = 1,275) and neighbourhood-level (*n* = 319) characteristics by fruit and vegetable consumption frequency, Chile, 2017^a^

	Fruit and vegetable consumption frequency			
	Total sample	Daily	Non-daily	p-value
**Individual-level characteristics**				
Number of participants	1,275	1,055 (82.7)	220 (17.3)	
Gender, n (%)^b^				
Female	785 (61.6)	903 (64.7)	102 (46.4)	< 0.001
Male	490 (38.4)	372 (35.3)	118 (53.6)	
Age in years, median (IQR) ^c^	50 (31–65)	52 (33–66)	38 (27–54)	< 0.001
Education, n (%)				
< Primary	135 (10.7)	116 (11.0)	20 (9.1)	0.36
Primary	580 (45.5)	468 (44.4)	112 (50.9)	
Secondary	393 (30.8)	331 (31.4)	62 (28.2)	
University	166 (13.0)	140 (13.3)	26 (11.8)	
**Neighbourhood-level characteristics, median (IQR)** ^c^				
Population density (number of people / km^**2**^)	9,809.5 (6,003.6–14,443.3)	9,751.5 (5,987.6–14,373.3)	10,403.2 (6,345.2–14,484.6)	0.31
Intraurban social environment index (z-score)	0.2 (-0.2,0.6)	0.2 (-0.2,0.6)	0.1 (-0.3,0.6)	0.031
**Total** ^d^	39.1 (18,63)	38.9 (18,63)	39.6 (18,62.6)	0.95
**Most Healthy**				
Fruit and vegetable stores	0 (0,0)	0 (0,0)	0 (0,0)	0.86
Fresh food retail	0 (0,1.5)	0 (0,1.6)	0 (0,1.5)	0.65
**Mixed**				
Supermarkets chains	2.8 (0,5.4)	2.8 (0,5.4)	2.9 (0,5.4)	0.74
Small food retail	4.8 (1.8,8.9)	4.6 (1.7,8.6)	5.3 (2.6,9.3)	0.08
Ready-for-consumption food retail	19.8 (8.4,40.1)	20.2 (8.4,40.4)	18.3 (8.5,36.7)	0.88
**Less Healthy**				
Candy and ice cream shops	0 (0,0)	0 (0,0.1)	0 (0,0.5)	1.00
Fast-food chains	0 (0,0)	0 (0,0)	0 (0,0)	0.98
Chain convenience stores	2.6 (0,7.9)	2.7 (0,7.9)	2.3 (0,7.9)	0.60

^a^ 3 cities combined

^b^ Chi-square test; ^c^ Mann-Whitney U Test

^d^ The row for ‘total’ includes all classifiable establishments *n* = 46,950 + unclassifiable establishments *n* = 1,450

IQR = Interquartile range

Among participants, 17.3% did not consume FV daily. Non-daily FV consumption was higher for males, younger people, and lower education. The availability of food establishments, categorised by healthiness, showed no significant variations based on consumption frequency (Table 4).

### Exploratory associations between FV consumption and community food environment

Most of the adjusted associations tested between food establishment density and odds of non-daily FV consumption were null (Table 5). The exception to this was for two food establishment categories that were the most prevalent in neighbourhoods: ‘small food retail’ (11% of the food establishments) and ‘ready for consumption’ (68% of the food establishments). Additionally, the association was strengthened after additional adjustment for potential confounders (comparing unadjusted model 1 to adjusted models 2 and 3). In the fully adjusted model, the odds of non-daily FV consumption were 20% and 17% higher with increases in the z-score of small food retail and ready-for-consumption food establishments, respectively (OR 1.20, 95% CI: 1.02, 1.40; and OR 1.17, 95% CI: 1.10, 1.37, results from model 3).

**Table 5:** Association between non-daily fruit and vegetable consumption and neighbourhood-level retail food environment, Chile, 2017 (*n* = 1,275)^a^

Food establishments density per area (count / km^2^)		Model 1	Model 2	Model 3
		OR (CI)		
Total ^b^		1.08 (0.94, 1.23)	1.09 (0.94, 1.26)	1.15 (0.97, 1.37)
Most healthy	Fruit and vegetable stores	0.94 (0.79, 1.10)	0.93 (0.78, 1.10)	0.94 (0.79, 1.12)
	Fresh food retail	1.07 (0.92, 1.23)	1.07 (0.92, 1.24)	1.10 (0.94, 1.28)
Mixed	Small food retail	1.15 (1.00, 1.31)	1.16 (1.01, 1.34)	1.20 (1.03, 1.40) ^**c**^
	Supermarkets chains	1.01 (0.87, 1.17)	1.01 (0.86, 1.18)	1.03 (0.88, 1.20)
	Ready-for-consumption food retail	1.08 (0.94, 1.23)	1.10 (0.95, 1.27)	1.17 (1.1, 1.35) ^**c**^
Less healthy	Candy and ice cream shops	0.98 (0.84, 1.15)	0.99 (0.84, 1.17)	1.02 (0.87, 1.20)
	Chain convenience stores	0.98 (0.84, 1.14)	0.96 (0.82, 1.13)	0.97 (0.82, 1.14)
	Fast-food chains	0.99 (0.85, 1.16)	1.0 (0.85, 1.16)	0.96 (0.80, 1.13)

^a^3 cities combined

Abbreviation: OR = odds ratio, CI = 95% confidence interval

Mixed effects logistic regressions with random effects at the neighbourhood-level. Outcome: Non-daily fruit and vegetable consumption (< 7 days a week). Key exposure: neighbourhood-level total and type specific food establishments density per area (km^2^)

Model 1: unadjusted. Model 2: adjusted by individual-level confounders age, gender and education. Model 3: adjusted by individual-level and area-level confounders population density, intraurban social environment index (z-score)

^b^ The row for ‘total’ includes all classifiable establishments *n* = 46,950 + unclassifiable establishments *n* = 1,450

^c^*P* ≤ 0.05

## Discussion

This study used a novel web data mining tool, utilising Google Cloud services, to identify and characterise the community food environment across three Chilean cities and investigated the association between community food environment and non-daily consumption of FV among 1,275 residents. We successfully identified, classified and georeferenced 46,950 food establishments across the three cities. Neighbourhoods with higher population density and more favorable social environments had higher density of food establishments. We also found that residing in neighbourhoods with higher densities of small food retail and ready-for-consumption foods was associated with higher odds of non-daily FV consumption.

Our study found that most food establishments were in the health-related category ‘mixed’ (signifying they sell healthier and unhealthier foods) and that there was a lack of healthy food stores, findings aligned with patterns previously observed in other Latin American countries [22, 27]. In particular, our work found the food establishment categories ‘small food establishments’ and ‘ready-for-consumption establishments’ were the most prevalent. The high prevalence of these types of establishments that sell UPF and high calorie ready-for-consumption meals, as opposed to establishments primarily selling healthy foods, may underlie insufficient FV intake among many Chileans [28] as well as escalating rates of overweight and obesity in Chilean urbanised areas over the past decade [29]. Notably, Chile leads the region in ultra-processed product sales and ranks second only to Peru in the increase in daily per capita retail sales of ultra-processed products also in the last decade [30]. In this context our findings provide subsidies for public policies for the development of public health policies designed to prevent both the displacement of fresh food markets by supermarkets and the shift in the food offerings of small food retailers, from traditional foods to ultra-processed products [16].

It should be noted that our study may provide a more pessimistic depiction of the community food environment than what actually exists, as our tool is unable to detect establishments that are not fixed points of food sales and, therefore, are not registered in Google’s database. Given that street markets/street food vendors play a crucial role in providing FV and fish in Chile [31], it is likely that the availability of outlets selling these healthy foods was higher.

The positive correlation we found between virtually all types of food retail and more favourable neighbourhood social environment also aligns with results observed in other Latin American countries [4, 22]. In Chile, the high availability of all types of foods and beverages may be contributing to the observed rise in obesity prevalence among individuals with higher socioeconomic status, who are experiencing faster increases in obesity rates compared to those in lower socioeconomic strata [29]. Beyond contextual differences, this pattern may be explained by differences in purchasing power. In Chile, evidence shows that the dietary share of UPFs increases with income, likely because higher-income individuals can afford both high- and low-quality foods, while lower-income individuals may have restricted access to UPFs due to their relatively higher cost in Latin America [32].

This paper presents a proof of concept for utilizing Google Cloud Services, specifically Google Earth, as a valuable tool for food environment surveillance in Latin America where usable food establishment data are scarce across large geographic areas [19]. Traditional methods for food environment data collection through direct field observations can pose significant challenges, especially in low-middle-income countries such as Chile, given their labour-intensive and expensive nature [33]. Additionally, relying solely on secondary data sources often necessitates validation studies [34], which can be a limiting factor in countries where comprehensive administrative databases are not readily available at the national level, as is the case in most Latin American countries [4]. Although our web tool method can still be time-consuming and expensive in part due to Google’s query fee structure (19), it represents a promising approach for researchers and policymakers seeking insights into food access and distribution in areas where such data are lacking, ultimately facilitating evidence-based strategies for improving community food environments and public health.

Our study has several significant strengths. (I) We compiled a census of almost 50,000 food establishments in cities where no primary or secondary data on community food environment exist. (II) We described the neighbourhood context where the food establishments are located (population density and social environment). (III) We linked food environment data to survey participants in Chile’s national health survey and examined associations between food environment and fruit and vegetable consumption.

However, our study also has noteworthy limitations. (I) We may have under- or over-identified food establishments due to their absence from Google’s map database or because we were unable to categorise them based on the establishments’ names or the Google-assigned food retailer type. Nevertheless, any resulting measurement error is likely to be fairly random. A previous validation study in two large Brazilian cities found that the tool’s overall accuracy was high, and that under- or over-reporting did not vary by neighbourhood socioeconomic status [19]. Although we did not replicate the validation study in the three Chilean cities, the use of the tool is justified because their demographic and commercial profiles—marked by large populations, high population density, and active retail and/or tourism sectors—closely resemble those of the Brazilian cities where the tool performed well [19]. (II) Google Earth is unable to detect street markets/street food vendors, which are an important source of healthy foods in Chile [31]. It is possible that the lack of an association reported in this study between the healthiest food establishment category and FV consumption, was due to the absence of data on informal food establishments. (III) There was a 4-year difference between the Google Earth data collection and dietary survey data and the community environment could have changed. (IV) Participants self-reported their FV consumption based only on a couple of questions in the survey, which may have presented a more positive scenario than the actual one, as most individuals reported daily consumption. A more detailed dietary assessment could have identified insufficient FV consumption despite some daily intake. Nevertheless, FV consumption is a key marker of healthy eating and non-daily FV intake aligned with expected patterns found in population based dietary surveillance (for example being higher among males, younger people, lower educated) [35].

## Conclusion

In conclusion, our study highlights significant disparities in the density of food establishments across Chilean neighbourhoods, characterised by a predominance of unhealthy outlets and a scarcity of healthier ones. Notably, nearly 20% of adults in our study did not consume a fruit or vegetable every day. We found that residing in neighbourhoods with higher densities of small food establishments and ready-for-consumption food establishments was associated with a higher likelihood of not consuming fruits and vegetables daily.

These findings underscore the need for targeted public policies aimed at promoting healthier food environments, reducing social inequalities in food access within Chilean cities, and increasing fruit and vegetable consumption. Policymakers in Chile could develop strategies to empower small retailers to maintain their traditional sales profile, focusing on non-ultra-processed foods. For example, implementing fiscal incentives—such as subsidies and tax reductions—would support small retailers that prioritise fresh and minimally processed foods. Additionally, strengthening supply chains by directly connecting small retailers with local producers could improve access to fresh food while reducing costs. These measures would enhance local food commerce, promote healthier food access, and help reduce social inequalities in food access and fruit/vegetable consumption in urban communities.

## Electronic supplementary material

Below is the link to the electronic supplementary material.

Supplementary Material 1

Supplementary Material 2

Supplementary Material 3

## Data Availability

This study used data from SALURBAL project. These data are not publicly available. The SALURBAL project welcomes queries from anyone interested in learning more about its dataset and potential access to them. To learn more about SALURBAL’s dataset, visit https://drexel.edu/lac/ or contact the project at salurbal@drexel.edu. The query script written used by this paper can be made available for academic research and non-commercial purposes and is provided ‘as-is’ upon request; please contact daniel.skaba@gmail.com.
