# Supplementary material for: Application of a geospatial query tool to characterise the community food environment and examine associations with dietary quality: evidence from three Chilean cities from the SALURBAL project
Source: BMC Public Health. 2025 Jul 3;25:2311. doi: 10.1186/s12889-025-23392-x (PMC12224401; doi:10.1186/s12889-025-23392-x)
Supplement: Supplementary file 1 — Supplementary Material 1 [file 12889_2025_23392_MOESM1_ESM.docx]

**Additional file 1.** Food establishment category description and rationale for assigning to a health group.

| **Food establishment category** | **Description and rationale for assigning to the healthy group** | **Types of food establishments included** |
| --- | --- | --- |
| **Most healthy** |  |  |
| Fruit and vegetable stores | Only fruit and vegetable stores. It is indisputably a healthy food environment marker. |  |
| Fresh food retail | Stores that primarily sell fresh or minimally processed food. Complementarily to FV stores, it can be considered a healthy food environment marker. | Meat, poultry & fish shops, dairy shops, stores selling grains and seeds minus "FV stores". |
| **Mixed** |  |  |
| Small food retail | Small grocery stores are traditional grocery stores in Latin American contexts. These stores sell healthier and unhealthier foods. | Small grocery stores, non-alcoholic beverage stores and minimarkets minus "Chain convenience stores". |
| Supermarkets | These businesses are large format food stores that sell all classes of foods frequently without a clear predominance of any type. These are commonly owned by a corporate chain and outlets are operated on a franchise basis. | Super and Hypermarkets. In this case, the names of supermarket chains present in Chile were searched together with the terms defined. |
| Ready-for-consumption food retail | Establishments selling ready-for-consumption food in a variety of formats. These establishments offer healthy and unhealthy foods. | Restaurants, bars, snack bars, and non-chain fast food (minus “Fast food chains”) |
| **Less healthy** |  |  |
| Convenience stores | Small commercial establishments that sell primarily ultra processed foods. It is indisputably an unhealthy food environment marker. | Convenience stores. In this case, the names of franchise convenience stores present in Chile were searched together with the terms defined. |
| Fast food chains | Only large fast-food chains. It is indisputably an unhealthy food environment marker. | Large fast-food chains. In this case, only the names of fast food chains present in Chile were searched. Fast food stores that are not big brands could not be separated from other types of restaurants, and were allocated in the “Ready-for-consumption” category. |
| Candy and ice cream shops | Establishments specialized in selling ultra processed desserts. Although it is not a consecrated category of analysis in retail food environment studies, it can be considered an unhealthy food environment marker. | Candy shops (in general or specialized, e.g. chocolate) and ice cream shops. |
