# Supplementary material for: Application of a geospatial query tool to characterise the community food environment and examine associations with dietary quality: evidence from three Chilean cities from the SALURBAL project
Source: BMC Public Health. 2025 Jul 3;25:2311. doi: 10.1186/s12889-025-23392-x (PMC12224401; doi:10.1186/s12889-025-23392-x)
Supplement: Supplementary file 2 — Supplementary Material 2 [file 12889_2025_23392_MOESM2_ESM.docx]

## Additional file 2. Inclusion flow chart.


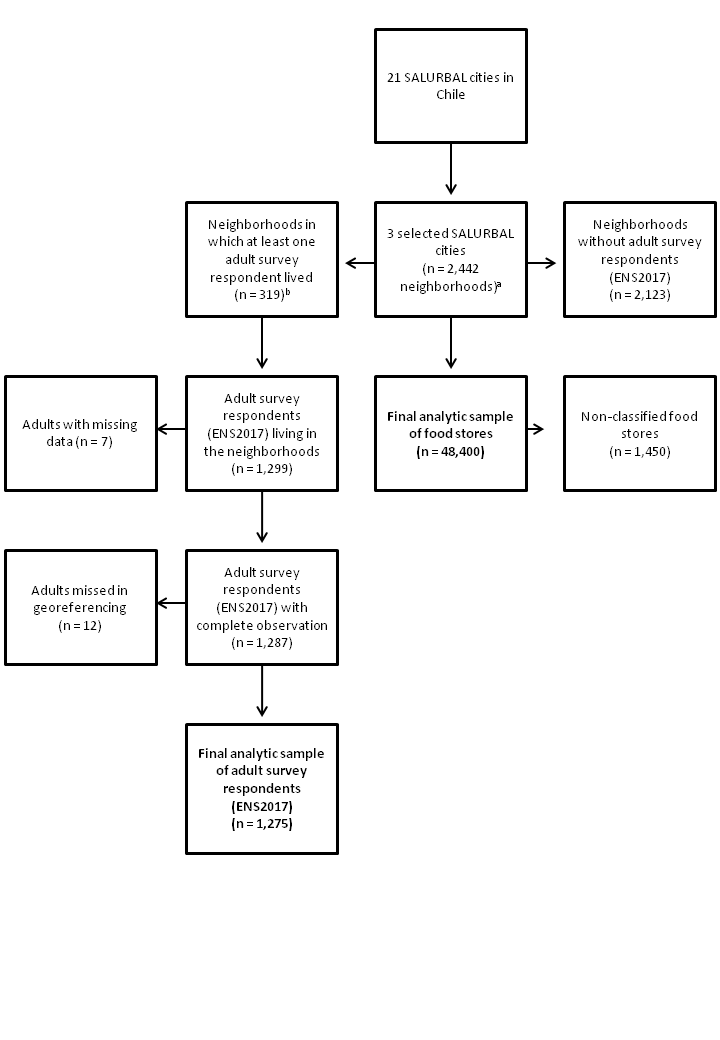


^a^Final analytic sample of neighbourhoods considered to describe the community retail food environment in the three cities. ^b^Final analytic sample of neighbourhoods considered to analyse the community retail food environment of the survey respondents.
