# Supplementary material for: Application of a geospatial query tool to characterise the community food environment and examine associations with dietary quality: evidence from three Chilean cities from the SALURBAL project
Source: BMC Public Health. 2025 Jul 3;25:2311. doi: 10.1186/s12889-025-23392-x (PMC12224401; doi:10.1186/s12889-025-23392-x)
Supplement: Supplementary file 3 — Supplementary Material 3 [file 12889_2025_23392_MOESM3_ESM.docx]

## Additional file 3. Neighbourhood food retail environment, Chile, 2017 (n = 2,442 neighbourhoods)^a^.

|  | Health-related category | | Establishment category | |
| --- | --- | --- | --- | --- |
|  | N | Percent (%) | N | Percent of total(%) |
| **Total** | 48,400 | 100.0 |  |  |
| **Categories** |  |  |  |  |
|  |  |  |  |  |
| **Most Healthy** | 1,524 | 3.14 |  |  |
| FV stores |  |  | 491 | 1.01 |
| Fresh food retail |  |  | 1,033 | 2.13 |
|  |  |  |  |  |
| **Mixed** | 40,535 | 83.74 |  |  |
| Small food retail |  |  | 5,210 | 10.76 |
| Supermarkets |  |  | 3,375 | 6.97 |
| Ready for consumption food retail |  |  | 31,950 | 66.01 |
|  |  |  |  |  |
| **Less Healthy** | 4,891 | 13.11 |  |  |
| Candy and ice cream shops |  |  | 812 | 1.68 |
| Fast-food chains |  |  | 463 | 0.96 |
| Chain convenience stores |  |  | 3,616 | 7.47 |
|  |  |  |  |  |
| **Unclassified** | 1,450 | 3.00 |  |  |

^a^3 cities combined.
